# Relative bioavailability of ertugliflozin tablets containing the amorphous form versus tablets containing the cocrystal form 

**DOI:** 10.5414/CP204212

**Published:** 2022-05-16

**Authors:** Vaishali Sahasrabudhe, Kyle Matschke, Haihong Shi, Anne Hickman, Angela Kong, Barbara Rodríguez Spong, Beverly Nickerson, Kapildev K. Arora

**Affiliations:** 1 Pfizer Inc., Groton, CT, and; 2 Pfizer Inc., Collegeville, PA, USA

**Keywords:** ertugliflozin, SGLT2, amorphous, cocrystal, bioavailability

## Abstract

Objectives: Ertugliflozin is a selective sodium-glucose cotransporter 2 inhibitor approved for the treatment of type 2 diabetes in adults. In its natural form, ertugliflozin exists as an amorphous solid with physicochemical properties that prevent commercial manufacture. The commercial product was developed as an immediate-release tablet, consisting of an ertugliflozin-L-pyroglutamic acid cocrystal of 1 : 1 molar stoichiometry as the active pharmaceutical ingredient. The ertugliflozin cocrystal may partially dissociate when exposed to high humidity for extended periods, leading to the formation of free amorphous ertugliflozin. Therefore, a study was conducted to estimate the relative bioavailability of ertugliflozin when administered in non-commercial formulated tablets containing the amorphous form vs. the cocrystal form. Materials and methods: In this phase 1, open-label, randomized, two-period, two-sequence, single-dose crossover study, 16 healthy subjects received 15 mg immediate-release ertugliflozin in its amorphous and cocrystal forms under fasted conditions, separated by a washout period of ≥ 7 days. Blood samples were collected post-dose for 72 hours to determine plasma ertugliflozin concentrations. Results: Mean ertugliflozin plasma concentration-time profiles were nearly superimposable following administration of the amorphous and cocrystal forms. The 90% confidence intervals for the geometric mean ratios for AUC_inf_ and C_max_ were wholly contained within the pre-specified criteria for similarity (70 – 143%), as well as the acceptance range for bioequivalence (80 – 125%). Most adverse events were mild in intensity. Conclusion: Any dissociation of ertugliflozin to the amorphous form that occurs in tablets containing the cocrystal will not have any clinically meaningful impact on the oral bioavailability of ertugliflozin.

**What is known about this subject**

- Ertugliflozin is a selective sodium-glucose cotransporter 2 inhibitor approved for the treatment of type 2 diabetes mellitus in adults.
- The commercial product was developed as an immediate-release tablet, consisting of an ertugliflozin-L-pyroglutamic acid cocrystal of 1 : 1 molar stoichiometry as the active pharmaceutical ingredient.
- During in vitro stability studies, the ertugliflozin cocrystal demonstrated a propensity to partially dissociate when exposed to high humidity for extended periods, leading to the formation of free amorphous ertugliflozin.

**What this study adds**

- This phase 1, open-label, randomized, two-period, two-sequence, single-dose crossover study evaluated the relative bioavailability of ertugliflozin when administered in non-commercial formulated tablets containing the amorphous form vs. the cocrystal form.
- Mean ertugliflozin plasma concentration-time profiles were nearly superimposable following administration of the amorphous and cocrystal forms.
- This study demonstrates that any dissociation of ertugliflozin to the amorphous form that occurs in tablets containing the cocrystal will not have any clinically meaningful impact on the oral bioavailability of ertugliflozin.

## Introduction 

Ertugliflozin, a selective inhibitor of sodium-glucose cotransporter 2 (SGLT2), is approved in the United States (US), European Union (EU), and other countries as an adjunct to diet and exercise to improve glycemic control in adults with type 2 diabetes mellitus (T2DM) [1, 2]. Ertugliflozin selectively inhibits SGLT2 in the kidney, and thereby limits renal glucose reabsorption, increases urinary glucose excretion, and reduces plasma glucose and glycated hemoglobin (HbA1c) levels [3]. In phase 3 clinical studies, once-daily doses of ertugliflozin 5 mg and 15 mg, alone or in combination with other antihyperglycemic agents, improved glycemic control, reduced systolic blood pressure and body weight, and was generally well tolerated in patients with T2DM [4, 5, 6, 7, 8, 9, 10, 11]. In addition to ertugliflozin approval as a standalone therapy, it has also received separate approvals as a fixed-dose combination of ertugliflozin with metformin [12, 13], and with sitagliptin (a dipeptidyl peptidase-4 inhibitor) [14, 15]. 

Ertugliflozin is a class I drug, according to the Biopharmaceutics Classification System, because it has high aqueous solubility and high membrane permeability [3, 16]. The absolute bioavailability is ~ 100% [16]. The highest ertugliflozin dose strength of 15  mg is completely soluble in ≤  250 mL of aqueous media over the pH range 1.2 – 6.8 (37 ± 1 °C), and ertugliflozin immediate-release (IR) tablets dissolve rapidly in vitro (≥ 85% of drug in tablet within 15 minutes) [2, 16]. Following oral administration, ertugliflozin is rapidly absorbed under fasted and fed conditions, with a median time to peak plasma concentration (t_max_) post-dose of ~ 1 hour and ~ 2 hours, respectively [3]. Administration of the ertugliflozin 15-mg tablet with food resulted in no meaningful effect on ertugliflozin area under the plasma concentration-time curve (AUC), but decreased peak concentrations (maximum observed plasma concentration, C_max_) by 29%. This effect on C_max_ is not clinically relevant and ertugliflozin can be administered without regard to food [17]. C_max_ and AUC from time 0 extrapolated to infinity (AUC_inf_), increase proportionally to dose over the dose range of 0.5 – 300 mg [18]. Terminal half-life (T_1/2_) ranges from 11 to 18 hours with steady-state concentrations achieved by 6 days after initiating once-daily dosing [18]. Ertugliflozin is primarily cleared via glucuronidation (86%), with a minor contribution from oxidative metabolism (12%), and little unchanged drug (1.5%) is excreted in urine [19]. 

**Figure 1.**
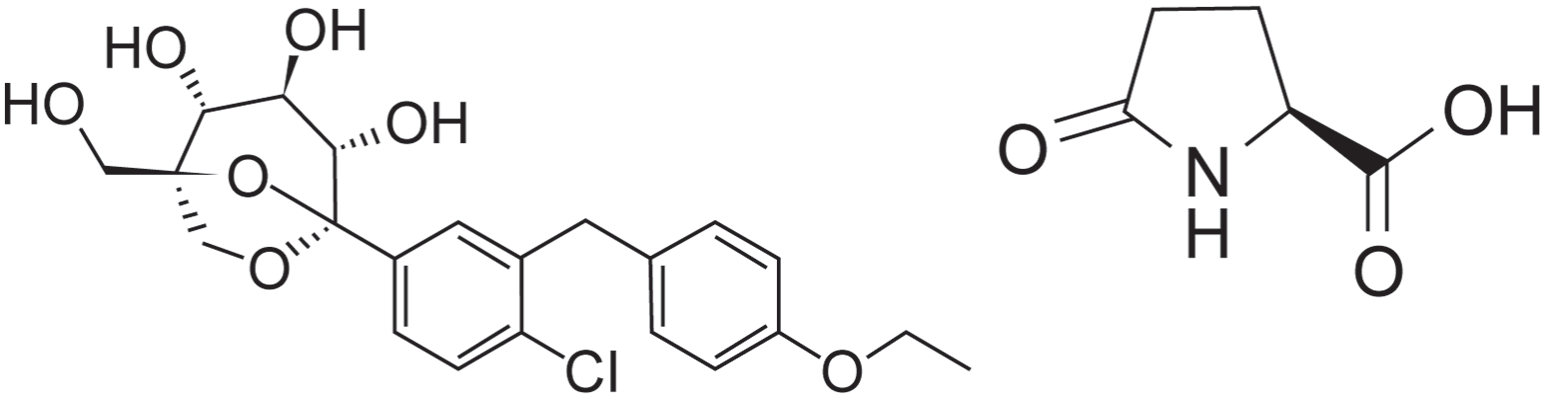
Chemical structure of ertugliflozin as a cocrystal with L-pyroglutamic acid.

In its natural form, ertugliflozin exists as an amorphous solid with undesirable physicochemical properties including high hygroscopicity and a propensity to convert into an oil. These properties prevent commercial-scale manufacture of the amorphous form with consistently high quality. As the functional groups in ertugliflozin are nonionizable under physiological pH conditions, options for salt formation are restricted. After extensive screening, an ertugliflozin cocrystal with L-pyroglutamic acid (L-PGA) was identified as a crystalline form for development. The ertugliflozin–L-PGA cocrystal was determined to be an anhydrous crystal form with a 1 : 1 molar stoichiometry of ertugliflozin and L-PGA. In contrast to the amorphous solid, the cocrystal is non-hygroscopic, with high melting point (~ 142 °C) and physicochemical stability at elevated temperatures and relative humidity, with no changes observed during any of the stability programs. It is also stable throughout the drug product manufacturing process and when stored in high density polyethylene (HDPE) bottles with desiccant and in aluminum foil blister packs [2]. The ertugliflozin drug product was developed as an IR tablet containing the cocrystal of ertugliflozin and L-PGA (Figure 1). The cocrystal has been used in all IR tablet dosage forms evaluated in clinical studies and commercial development. However, it was observed that the ertugliflozin cocrystal in the drug product can dissociate to the amorphous free form when the drug product was exposed to high humidity conditions in open dish accelerated stability, or during in-use stability (bottles opened and closed daily to simulate patient use) studies [20]. Amorphous content up to 27% was measured by FT-Raman in a 26 week in-use development stability study performed at 30 °C/75% relative humidity (RH). Although amorphous ertugliflozin was observed in the in-use samples, no significant changes were observed in the dissolution profile, and all other tests met acceptance criteria. In addition, in vitro tests showed that the amorphous form and the ertugliflozin–L-PGA cocrystal have similar high solubility that is pH-independent. 

To further assess the impact of ertugliflozin–L-PGA cocrystal dissociation on the bioperformance of the drug product, a clinical study was conducted. This phase 1, open-label, randomized, two-period, two-sequence, single-dose crossover study estimated the relative bioavailability of the amorphous form to the cocrystal form of ertugliflozin in healthy adult subjects. The safety and tolerability of a single oral dose of ertugliflozin when administered as tablets containing the amorphous form, or the cocrystal form, were also assessed. 

## Materials and methods 

### Study design 

This phase 1, open-label, randomized, two-period, two-sequence, single-dose crossover study was conducted in healthy subjects at the Pfizer Clinical Research Unit (CRU) in Brussels, Belgium. The study was compliant with the ethical principles of the Declaration of Helsinki and all International Conference on Harmonisation Good Clinical Practice guidelines. The final protocol and informed consent documentation for the study were reviewed and approved by the Comite d’Ethique Hospitalo-Facultaire Erasme-ULB Institutional Review Board (Brussels, Belgium), and all subjects provided signed and dated informed consent. 

**Figure 2.**
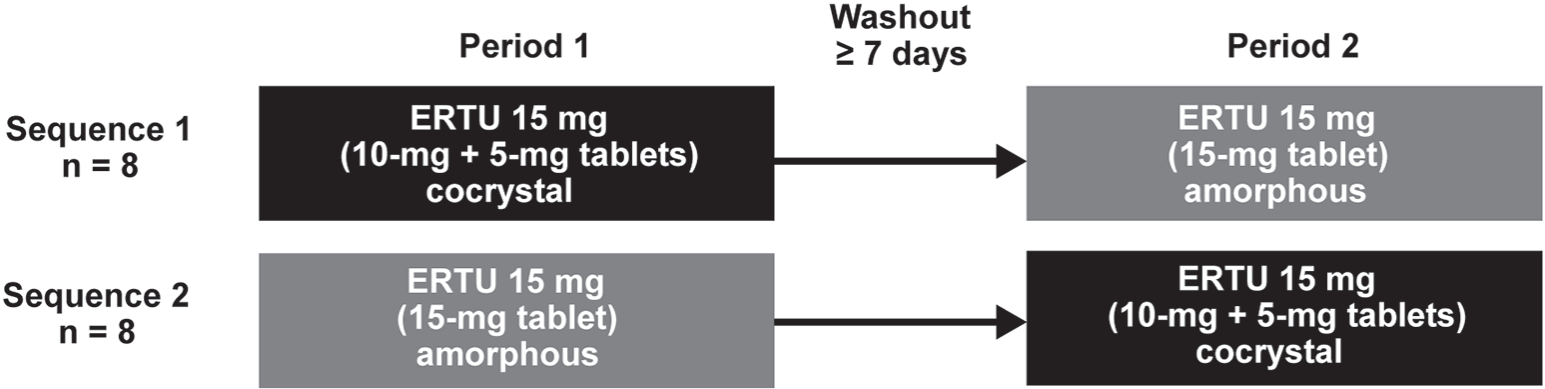
Treatment sequence for the study. ERTU = ertugliflozin.

The study consisted of a screening visit and 2 study treatment periods, with screening occurring within 28 days of the first dose of study treatment. A total of 16 healthy subjects (n = 8 per sequence) were enrolled in this study. Eligible subjects were admitted to the CRU on day 0. During the treatment periods, the subjects received a 15-mg dose of the amorphous form of ertugliflozin (test treatment), administered as one 15-mg tablet, or a 15-mg dose of the cocrystal form of ertugliflozin (reference treatment), administered as one 10-mg tablet and one 5-mg tablet, according to 1 of 2 treatment sequences (Figure 2). The ertugliflozin IR formulations used in the study were not the commercial formulation. Treatments were administered following an overnight fast of ≥ 8 hours, and treatment periods were separated by a washout period of ≥ 7 days. 

### Subjects 

Eligible subjects were healthy males and females aged 18 – 55 years with a body mass index 17.5 – 30.5 kg/m^2^ and a total body weight > 50 kg, who had provided a signed and dated informed consent form. Female subjects were either of non-childbearing potential or agreed to use accepted methods of contraception if they were of childbearing potential. “Healthy” was defined as having no clinically relevant abnormalities identified by a detailed medical history, full physical examination, including blood pressure and pulse rate measurement, 12-lead electrocardiogram (ECG), and clinical laboratory tests. 

Exclusion criteria included subjects showing evidence, or having a history, of clinically significant hematologic, renal, endocrine, pulmonary, gastrointestinal, cardiovascular, hepatic, psychiatric, neurologic, or allergic disease; any clinically significant malabsorption condition; a positive urine screen for drugs of abuse or recreation; history of alcohol abuse or binge drinking, and/or any other illicit drug use or dependence within 6 months of screening; an estimated glomerular filtration rate < 90 mL/min/1.73m^2^, based on the four-variable Modification of Diet in Renal Disease equation [21]; being pregnant or breastfeeding; using prescription or non-prescription drugs (except hormonal methods of birth control), vitamins, and dietary supplements within 7 days or 5 half-lives (whichever was longer) prior to the first dose of the study medication; and having any known hypersensitivity or intolerance to any SGLT2 inhibitor. 

### Pharmacokinetic assessments 

Serial blood samples (4 mL) for pharmacokinetic (PK) analysis of ertugliflozin were collected pre-dose (0 hour), and 0.25, 0.5, 1, 1.5, 2, 3, 4, 8, 12, 24, 48, and 72 hours after administration in each treatment period. Subjects were discharged from the CRU after collection of the 24-hour PK sample in each treatment period and returned to the CRU in an outpatient setting for collection of the 48- and 72-hour PK samples. Samples were centrifuged at ~ 1,700 x g for 10 minutes at 4 °C and then stored at –20 °C within 1 hour of collection. 

Plasma samples were analyzed for ertugliflozin concentrations at WuXi AppTec (Shanghai, China) using a validated, sensitive, and specific high-performance liquid chromatography–tandem mass spectrometry method, described previously [22]. Calibration standard responses were linear over the range of 0.5 – 500 ng/mL, using a weighted (1/concentration^2^) linear least squares regression. The lower limit of quantification (LLOQ) for ertugliflozin was 0.5 ng/mL. The between-day assay accuracy, expressed as percent relative error, for quality control (QC) concentrations, ranged from –4.3 to 2.4% for the low, medium, and high QC samples. Assay precision, expressed as the between-day percent coefficient of variation (%CV) of the mean estimated concentrations of QC samples was ≤ 3.6% for low (1.25 ng/mL), medium low (12.5 ng/mL), medium high (250 ng/mL), and high (400 ng/mL) concentrations. 

### Safety assessments 

Safety assessments for adverse events (AEs) were conducted from screening throughout the duration of study participation. Subjects had a follow-up phone call 14 ± 3 days after treatment administration in period 2 to assess for AEs including serious adverse events. Medical Dictionary for Regulatory Activities (MedDRA, version 17.0) coding was applied. 

### Statistical analysis 

Ertugliflozin PK parameters, for plasma samples, were calculated for each subject for each treatment using noncompartmental analysis of plasma concentration-time data. C_max_, t_max_, AUC from time 0 to time of last quantifiable concentration (AUC_last_), AUC_inf_, and T_1/2_ were summarized descriptively by treatment. Actual sample collection times were used, and samples below the LLOQ were set to 0, for the PK analysis. PK parameters were calculated using Pfizer-validated software, electronic noncompartmental analysis (eNCA, version 2.2.4). 

The PK concentration population included all treated subjects for whom at least 1 PK sample was taken after drug administration. The PK parameter analysis population included all treated subjects for whom at least 1 PK parameter was calculated. Natural log-transformed AUC_inf_, AUC_last_, and C_max_ of ertugliflozin were analyzed using a mixed-effects model with sequence, period, and treatment as fixed effects and subject within sequence as a random effect. The adjusted mean differences and 90% confidence intervals (CIs) for the differences obtained from the model were exponentiated to provide estimates of the ratio of adjusted geometric means (GMR) (test : reference (amorphous : cocrystal)) and 90% CI for the ratio. Bioavailability of the formulations was to be considered similar if the 90% CIs for the GMR for both AUC_inf_ and C_max_ fell wholly within 70 – 143%. A sample size of ~ 16 subjects was required to provide 96% power so that the 90% CI for the GMR fell within the acceptance region for each of the ertugliflozin PK parameters (AUC_inf_ and C_max_)_._ Consequently, this study had at least 92% power overall to demonstrate similarity of the amorphous form to the cocrystal form of ertugliflozin, with respect to C_max_ and AUC_inf_, where overall study power was based on the product of the individual powers of the parameters of interest. 

## Results 

### Study subjects 

A total of 16 subjects were enrolled in the study; 15 subjects completed the study, while 1 subject discontinued following their treatment in period 1, and prior to commencing period 2, due to experiencing an AE deemed unrelated to the study. All 16 subjects received the amorphous form and 15 subjects received the cocrystal form of ertugliflozin. The demographic characteristics of the study population are shown in Table 1. Equal numbers of male and female subjects were included, with 15 of 16 self-reporting as White. 

### Pharmacokinetic assessments 

**Figure 3.**
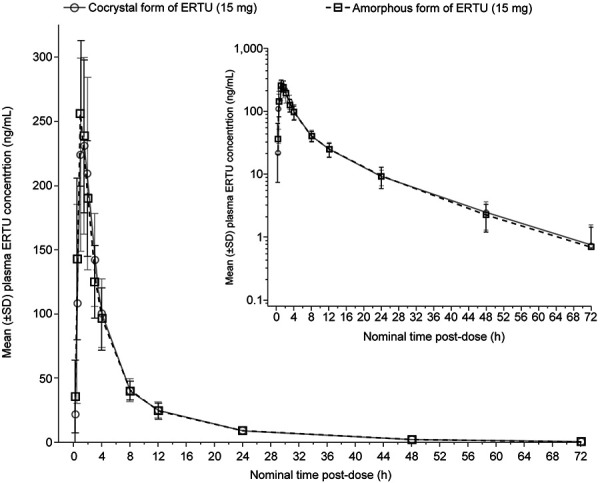
Mean ± SD plasma ertugliflozin concentration-time profiles. Main panel is linear scale; inset panel is semilogarithmic scale. Values below the LLOQ (0.5 ng/mL) were set to 0 for analysis. Summary statistics were not calculated for time points with no observations above the LLOQ. ERTU = ertugliflozin; LLOQ = lower limit of quantification; SD = standard deviation.

Mean ertugliflozin plasma concentration-time profiles were nearly superimposable following administration of the amorphous and cocrystal forms under fasted conditions (Figure 3). A descriptive summary of plasma ertugliflozin PK parameter values is provided in Table 2. The median t_max_ values for the amorphous form and the cocrystal form of ertugliflozin (1.00 hour and 1.50 hours, respectively) and the mean T_1/2_ values (11.7 hours and 12.4 hours, respectively) were similar between the treatment groups. 

**Figure 4.**
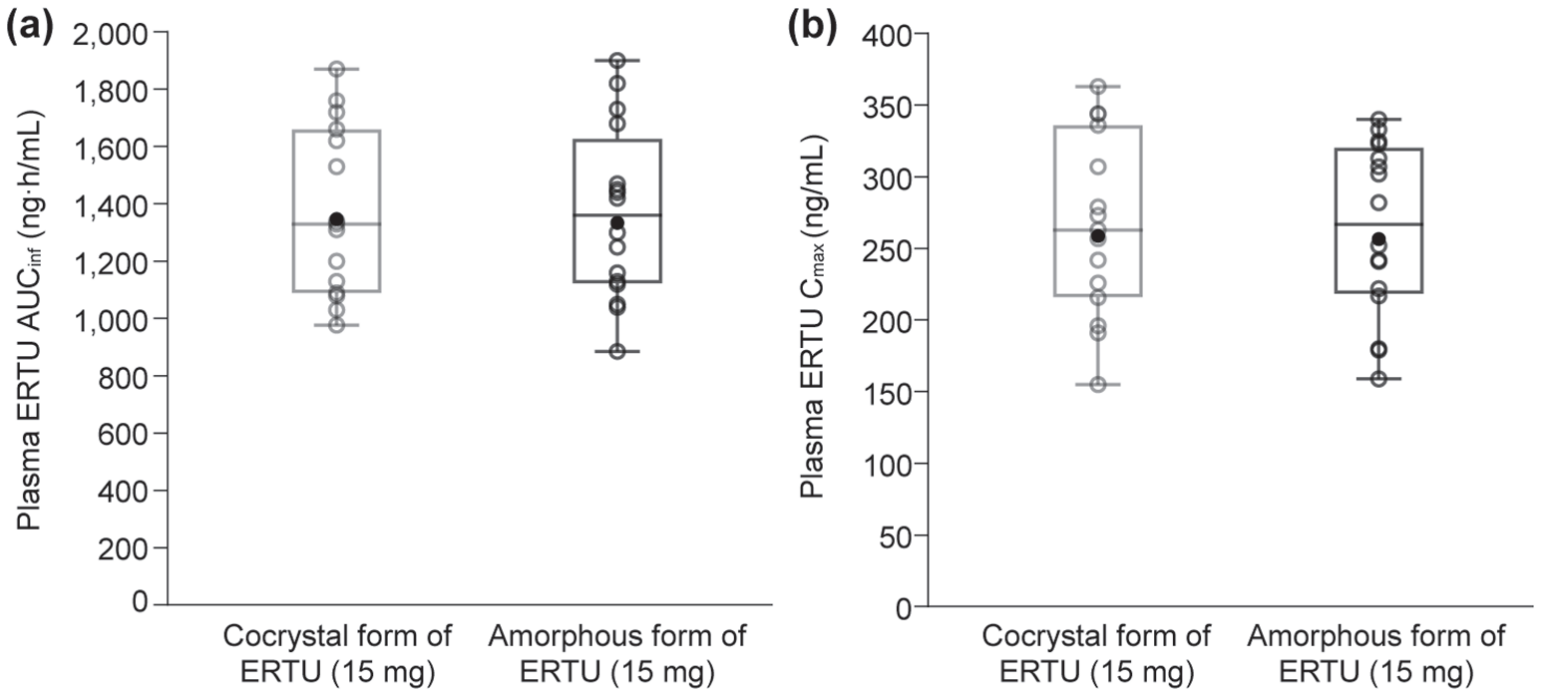
Individual values and geometric means of plasma ertugliflozin (a) AUC_inf_ and (b) C_max_. Closed circles represent geometric means and open circles represent individual subject values. Box plot provides median and 25%/75% quartiles with whiskers to the last point within 1.5× the interquartile range. AUC_inf_ = area under the plasma concentration-time curve from time 0 extrapolated to infinite time; C_max_ = maximum observed plasma concentration; ERTU = ertugliflozin.

The results of statistical comparisons are summarized in Table 3. The GMR (amorphous : cocrystal) was 98.70% (90% CI: 95.44 – 102.06%) for AUC_inf_ and 98.32% (90% CI: 92.23 – 104.81%) for C_max_. The 90% CIs for the GMRs for AUC_inf_ and C_max_ were contained within the protocol pre-specified criteria for similarity (70 – 143%). Additionally, the 90% CIs for AUC_inf_ and C_max_ were also wholly contained within the acceptance range for bioequivalence (80 – 125%). Individual values and geometric means of ertugliflozin AUC_inf_ and C_max_ for both the amorphous and cocrystal formulations are shown in Figure 4. 

### Safety 

No deaths, serious AEs, severe AEs, temporary discontinuations, or dose reductions due to AEs were reported during the study. One subject discontinued the study after experiencing an AE (an influenza-like illness of moderate intensity), which was not considered to be related to the study drug. This subject received the amorphous form of ertugliflozin in period 1 and did not participate in period 2. The number of subjects that experienced treatment-emergent AEs was similar for both ertugliflozin formulations, as was the number of subjects that experienced AEs considered to be related to treatment (Supplementary Table 1). Nine treatment-related AEs were reported in subjects receiving the cocrystal form, and 7 treatment-related AEs were reported in subjects receiving the amorphous form. The majority of AEs (17 of 24 events) were mild in intensity; the remaining seven events, reported in 5 subjects, were moderate in intensity. The most frequently reported AEs by preferred term were headache (6 subjects), nausea (5 subjects), and fatigue (4 subjects), without any noticeable pattern of difference in AEs between administration of the amorphous and cocrystal forms. 

## Discussion 

Ertugliflozin was developed as an anhydrous cocrystal of 1 : 1 molar stoichiometry with L-PGA. In this clinical study, the bioperformance of ertugliflozin was assessed upon oral administration of tablets containing the cocrystal form and tablets containing the amorphous form. The results of this study indicated that cocrystal and amorphous forms of ertugliflozin were bioequivalent upon oral administration in healthy adult subjects, under fasted conditions. A single oral dose of 15 mg was used in this study as it was the highest dose that was evaluated in phase 3 studies and the highest dose approved for therapeutic use. Results from the 15-mg dose are applicable to lower dose strengths based on the dose-proportional PK of ertugliflozin over the therapeutic dose range and the composition of the 15-mg tablet being proportionally similar to that of the 5-mg tablet. Additionally, as this study compared 100% amorphous tablets to tablets containing the cocrystal, any degree of dissociation of ertugliflozin to the amorphous form that occurs in tablets containing the cocrystal will not have any clinically meaningful impact on the oral bioavailability of ertugliflozin. 

Ertugliflozin is formulated in the cocrystal form due to the challenges generally associated with manufacturing amorphous drugs, particularly their lack of robustness, which could lead to varying product quality during stages of processing. Moreover, developing the amorphous form of ertugliflozin provides no solubility advantage, as might otherwise be the case for poorly water-soluble drugs [20], with both the cocrystal and amorphous forms of ertugliflozin having similarly high aqueous solubility (~ 0.76 mg/mL, pH independent) [23]. Other cocrystal active pharmaceutical ingredients, however, have been described to dissociate under certain conditions, potentially leading to compromised absolute oral bioavailability [24]. Drivers of cocrystal dissociation have been suggested to include the inversion of relative free energies of the cocrystal and individual cocrystal components (cocrystal coformers) at high temperature [24], cocrystal dissolution and subsequent precipitation of coformers, and loss of coformer upon heating [25]. During in vitro stability studies, the ertugliflozin–L-PGA cocrystal drug product demonstrated a propensity to partially dissociate into the amorphous form of ertugliflozin at high humidity in the presence of excipients [26]. In studies conducted to help understand the cocrystal dissociation mechanism, the predicted pH-solubility curve for the cocrystal did not intersect with either ertugliflozin or L-PGA and therefore it was concluded that the cocrystal does not have a pH of maximum solubility (pH_max_), often observed with ionic salts. The solubility ratio of the cocrystal and the amorphous ertugliflozin increased exponentially as a function of pH, with a greater degree of supersaturation of the amorphous free form to cocrystal at pH > 5.0. A binary excipient compatibility study to investigate the influence of the physicochemical properties of excipients on the physical stability of the cocrystal showed a good correlation between excipient properties (at least pH and hygroscopicity) and cocrystal dissociation in the formulations. In general, hygroscopic excipients that create a pH > 5.0 microenvironment induced dissociation of ertugliflozin–L-PGA cocrystal to amorphous ertugliflozin in its free form [26]. However, ertugliflozin exhibits high aqueous solubility regardless of solid-state form. Indeed, in vitro dissolution testing of ertugliflozin 15 mg IR tablets containing 100% cocrystal ertugliflozin and 100% amorphous ertugliflozin showed that both cocrystal and amorphous tablets dissolve very rapidly (≥ 85% dissolved in 15 minutes; data on file). The ertugliflozin cocrystal is stable throughout the drug product manufacturing process and when stored in HDPE bottles with desiccant and in aluminum foil blister packs [2]. However, if cocrystal dissociation occurs, it would not be expected to have any clinically meaningful effect on the bioperformance of ertugliflozin tablets. 

In line with previous phase 3 studies that have shown ertugliflozin to be well tolerated [4, 5, 6, 7, 8, 9, 10, 11], a single dose of 15-mg ertugliflozin prepared as tablets containing the amorphous or cocrystal form was also generally safe and well tolerated in healthy subjects, with only mild to moderate AEs reported during the study. 

## Conclusion 

This clinical study demonstrated that ertugliflozin–L-PGA cocrystal dissociation in tablets does not affect the oral bioavailability of the drug. Tablets containing the amorphous and cocrystal forms of ertugliflozin showed bioequivalence in healthy subjects under fasted conditions. The two forms of ertugliflozin were generally safe and well tolerated. These data support the development of ertugliflozin as a cocrystal-containing tablet formulation. 

## Ethnicity 

Previously conducted population pharmacokinetic analyses of ertugliflozin have suggested no dose modification of ertugliflozin is required on the basis of race/ethnicity. 

## Data sharing 

Upon request, and subject to certain criteria, conditions, and exceptions (see https://www.pfizer.com/science/clinical-trials/trial-data-and-results for more information) Pfizer will provide access to individual de-identified participant data from Pfizer-sponsored global interventional clinical studies conducted for medicines, vaccines, and medical devices (1) for indications that have been approved in the US and/or EU or (2) in programs that have been terminated (i.e., development for all indications has been discontinued). Pfizer will also consider requests for the protocol, data dictionary, and statistical analysis plan. Data may be requested from Pfizer trials 24 months after study completion. The de-identified participant data will be made available to researchers whose proposals meet the research criteria and other conditions, and for which an exception does not apply, via a secure portal. To gain access, data requestors must enter into a data access agreement with Pfizer. 

## Acknowledgment 

Medical writing support was provided by Jamal Khan, PhD, of Engage Scientific Solutions (Sydney, Australia) and was funded by Pfizer Inc., New York, NY, USA and Merck Sharp & Dohme Corp., a subsidiary of Merck & Co., Inc., Kenilworth, NJ, USA. 

## Authors’ contributions 

All authors critically reviewed the draft manuscript and approved the final version of the manuscript for publication. All authors were involved in the conception/design of the study. All authors were involved in data analysis and interpretation of the data. 

## Funding 

This study was sponsored by Pfizer Inc., New York, NY, USA, and Merck Sharp & Dohme Corp., a subsidiary of Merck & Co., Inc., Kenilworth, NJ, USA. 

## Conflict of interest 

Vaishali Sahasrabudhe, Kyle Matschke, Haihong Shi, Anne Hickman, Angela Kong, Barbara Rodríguez Spong, Beverly Nickerson, and Kapildev K. Arora are employees of Pfizer Inc., and may own shares/stock options in Pfizer Inc. 

**Table 1.** Summary of subject demographic characteristics.

Characteristic	Study population (N = 16)
Gender, n	
Male	8
Female	8
Age, years	
Mean	31.2
SD	10.5
Range	20 – 53
Race, n	
White	15
Black	1
Weight, kg	
ean	73.2
SD	12.4
Range	58.7 – 98.0
BMI (kg/m^2^)	
Mean	25.3
SD	2.8
Range	21.3 – 30.2
Height, cm	
Mean	169.6
SD	7.9
Range	156 – 182

BMI = body mass index; SD = standard deviation.

**Table 2.** Descriptive summary of plasma ertugliflozin PK parameter values.

PK parameter	Amorphous form of ERTU (15 mg)	Cocrystal form of ERTU (15 mg)
N, n	16, 16	15, 15^a^
AUC_inf_, ng·h/mL	1,334 (22)	1,347 (22)
AUC_last_, ng·h/mL	1,315 (22)	1,329 (22)
C_max_, ng/mL	256.6 (25)	258.8 (25)
t_max_, h	1.00 (1.00 – 1.50)	1.50 (1.00 – 3.00)
T_1/2_, h	11.71 ± 2.37	12.37 ± 2.74

Values are geometric mean (geometric %CV) except: median (range) for t_max_ and arithmetic mean (SD) for T_1/2_. ^a^One subject discontinued from the study following treatment with the amorphous form. AUC_inf_ = area under the plasma concentration concentration-time curve from time 0 extrapolated to infinite time; AUC_last_ = area under the plasma concentration concentration-time curve from time 0 to time of last quantifiable concentration; C_max_ = maximum observed plasma concentration; CV = coefficient of variation; ERTU = ertugliflozin; N = number of subjects in the treatment group and contributing to the summary statistics; n = number of subjects with reportable T_1/2_ and AUC_inf_; PK = pharmacokinetics; SD = standard deviation; T_1/2_ = terminal half-life; t_max_ = time to maximum observed plasma.

**Table 3.** Statistical summary of treatment comparisons for plasma ertugliflozin PK parameter valuesa.

PK parameter	Adjusted geometric means		ERTU Amorphous : cocrystal GMR^a^	90% CI for GMR^a^
	Amorphous form of ERTU (test)	Cocrystal form of ERTU (reference)		
AUC_inf_, ng·h/mL	1,334	1,352	98.70	95.44 – 102.06
C_max_, ng/mL	256.6	261.0	98.32	92.23 – 104.81

^a^The ratios and 90% CIs are expressed as percentages. AUC_inf_ = area under the plasma concentration–time curve from time 0 extrapolated to infinite time; CI = confidence interval; C_max_ = maximum observed plasma concentration; ERTU = ertugliflozin; GMR = geometric mean ratio; PK = pharmacokinetic.

## Supplemental material

Supplemental material Supplementary Table 1.
