# Supplementary material for: Relative bioavailability of ertugliflozin tablets containing the amorphous form versus tablets containing the cocrystal form
Source: Int J Clin Pharmacol Ther. 2022 May 16;60(7):317–26. doi: 10.5414/CP204212 (PMC9238437; doi:10.5414/CP204212)
Supplement: Supplemental material [file intjclinpharmacol-60-317-S01.pdf]

Supplementary table 1. Summary of treatment-emergent AEs

	Amorphous form of ERTU (15 mg)		Cocrystal form of ERTU (15 mg)	
	All causality	Treatment-related	All causality	Treatment-related
Subjects evaluable for AEs	16	16	15	15
Number of AEs	11	7	13	9
Subjects with AEs	7	5	8	6
Subjects with serious AEs	0	0	0	0
Subjects with severe AEs	0	0	0	0
Subjects discontinued due to AEs	1	0	0	0
Incidence of treatment-emergent AEs	All causality	Treatment-related	All causality	Treatment-related
Gastrointestinal disorders	2	2	4	2
Abdominal pain	0	0	1	0
Nausea	2	2	3	2
Vomiting	1	1	0	0
General disorders and administration-site conditions	3	2	4	4
Chest pain	1	1	0	0
Fatigue	1	1	3	3
Feeling hot	0	0	1	1
Influenza-like illness	1	0	0	0

Injury, poisoning, and procedural complications	2	0	1	0
Thermal burn	1	0	0	0
Tooth fracture	0	0	1	0
Wound	1	0	0	0
Nervous system disorders	2	2	4	3
Headache	2	2	4	3
Skin and subcutaneous tissue disorders	1	0	0	0
Erythema	1	0	0	0
Total preferred term events	11	7	13	9

Includes all data collected since the first dose of study drug.

MedDRA (version 17.0) coding dictionary applied.

AE = adverse event; MedDRA = Medical Dictionary for Regulatory Activities.
